# Work and income changes after cancer in rural China: A cross‐sectional survey

**DOI:** 10.1002/cam4.2627

**Published:** 2019-10-25

**Authors:** Mingzhu Su, Nan Zhang, Yuanchu Cai, Jialin Wang, Roger Anderson, Nengliang Yao, Xiaojie Sun

**Affiliations:** ^1^ School of Health Care Management (Key Lab of Health Economics and Policy, National Health Commission) Shandong University Jinan Shandong China; ^2^ Center for Cancer Control and Policy Research School of Health Care Management Shandong University Jinan Shandong China; ^3^ Shandong Cancer Hospital and Institute Shandong First Medical University and Shandong Academy of Medical Sciences Jinan Shandong China; ^4^ Department of Public Health Sciences University of Virginia Charlottesville VA USA; ^5^ University of Virginia Cancer Center Charlottesville VA USA

**Keywords:** cancer survivors, China, return to work, rural, work disability

## Abstract

**Background:**

The present study aimed to first describe the work‐related outcomes of cancer survivors and to then identify those characteristics that influenced the decision to stop working in rural China.

**Methods:**

We assessed 752 cancer survivors (residents of rural areas, working at the time of diagnosis, >1 year since completing treatment) from the cross‐sectional study “China Survey of Experiences with Cancer”. Participants reported changes in employment status, income, and the ability to perform physical jobs due to cancer, as well as the work‐related outcomes of their informal caregivers. Logistic regression analyses were used to examine the association between sociodemographic characteristics, cancer characteristics, and changes in work (ie, continue to work vs not working).

**Results:**

The participants were largely farmers (96%), women (56%), younger than 65 years old (69%), and diagnosed with colorectal (31%) and breast cancer (31%). Thirty‐nine percent reported reducing working hours, and 40% reported stopping work altogether. Approximately 7% of informal caregivers also stopped working in order to take care of those diagnosed with cancer. Thirty‐three percent of cancer survivors and 5% of their informal caregivers had no source of income following treatment. Controlling for other variables, lower educational attainment, physical limitations in work, and different cancer sites were significantly associated with ending employment in both men and women, while among men specifically, we observed that older age, being unmarried, and being diagnosed at later stages were significantly associated with an end to working.

**Conclusion:**

Rural cancer survivors are at a high risk for stopping work after completing treatment, and many survivors and their caregivers experience poor work‐related outcomes and economic hardship. These results highlight the importance of paying attention to the work experiences of cancer survivors in rural China.

## Graphical abstract

The present study aimed to first describe the long‐term effects of cancer diagnosis and treatment on the work status of cancer survivors, and to then identify those characteristics that influenced the decision to stop working in rural China

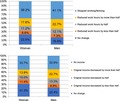

## 1 BACKGROUND

Of the estimated 18.1 million new cancer cases and 9.6 million deaths from cancer worldwide in 2018, approximately 24% of all cases occurred in China, and 30% of all cancer deaths occurred in China alone, with lung cancer being the most common.^1^, ^2^ Stomach, esophageal, liver, breast, and colorectal cancer were also commonly diagnosed in China.^2^ Due to improved detection methods and more effective treatments as well as an overall increase in longevity, the number of cancer survivors continues to increase.^3^, ^4^ Thus, it is increasingly important to understand the lasting effects of cancer survivorship on these individuals' physical, psychological, and social well‐being in China.

The ability to return to work actively is important in maintaining family and social roles. Return to work and work ability after cancer should therefore be regarded as critical topics within psychosocial and cancer survivorship research. A meta‐analysis of unemployment among cancer survivors found that cancer survivors were 1.37 times more likely to experience long‐term unemployment than the general population.^5^ A previous study on employment issues showed that most cancer patients, while more likely than the general population to experience unemployment, are able to return to work after treatment, and it also revealed certain risk factors, such as patients' characteristics and work environment.^6^ Janet de Moor et al found that cancer survivors who received a treatment summary were more likely to talk about employment with health‐care providers than those who did not.^7^ Another study documented the effectiveness of various programs initiated by employers, health‐care providers, and legislation in helping survivors return to work in developed countries.^8^ In China, a large body of cancer disparity research projects has focused on identifying the population characteristics linked to cancer incidence, treatment, cost, and death.^9^, ^10^, ^11^ Few studies, though, have examined the link between the cancer survivors' population characteristics and work‐related issues, especially among rural cancer survivors in China. There is a pressing need for research of this kind since of the over 4.29 million new cancer cases in China in 2015, nearly half of them occurred in rural areas.^2^

Studies have shown that cancer and cancer treatments might have more lasting effects on employment among rural Chinese survivors of cancer than their urban counterparts.^12^, ^13^, ^14^, ^15^, ^16^, ^17^, ^18^, ^19^, ^20^ Rural residents often face greater challenges and limitations in finding employment as a result of China's household registration system (*hukou*), which has separated rural residents from urban populations.^12^ Rural workers usually have no choice but to work in agriculture or labor‐intensive industries on short‐term and informal contracts.^13^ It may therefore be more challenging to return to work after cancer in rural settings where physically demanding jobs are more common.^14^ Rural residents also usually have fewer retirement benefits and less legal protection than urban employees.^15^ Rural residents are mostly insured by the New Rural Cooperative Medical System, which has less coverage and higher coinsurance rates than the insurance that covers urban employees.^16^ Rural patients also have more restricted access to health care due to the shortage of cancer care resources and qualified providers.^17^, ^18^ People living in rural areas are also more likely to be diagnosed at more advanced stages of cancer,^19^ and health shock has become a significant cause of poverty or a family returning to poverty.^20^

Understanding rural cancer survivors' work‐related problems as a result of cancer diagnosis and treatment is essential in assessing their health‐related quality of life and financial hardship, and in developing the interventions of rehabilitative and occupational care after primary treatments. Prior studies found that there was a gender wage difference among rural workers worldwide, and the difference was influenced by the combination of human capital difference, compensatory wage difference, labor market segmentation, and labor market discrimination.^21^, ^22^ Luo constructed a theoretical analysis framework of gender wage differentials for rural workers in China.^23^ In his research, he described the sociodemographic, human capital, and employment characteristics that result in wage differences. Despite the importance of work‐related issues among rural cancer survivors, few studies have paid attention to their ability to return to work.^24^, ^25^ The literature on cancer‐related employment and work‐related changes, suggests that the employment status of cancer survivors was affected by four groups of factors.^26^, ^27^, ^28^ Sociodemographic factors include their age, marital status, and educational level. Cancer stage, cancer site, time since diagnosis, fatigue, and physical symptoms are health‐related factors affecting employments status. Psychosocial determinants are social support, depression, and anxiety. Lastly, employment factors include the type of work, individual meaning of work, and the relationship with coworkers and managers. To fill this gap in data, this study aimed to describe work‐related outcomes among rural cancer survivors and their family caregivers in China, a previously overlooked population. The secondary goal was to identify the various relationships between patient sociodemographics, cancer characteristics, and an end in employment among cancer survivors.

## 2 METHODS

### 2.1 Study design

We designed the “China Survey of Experiences with Cancer” for cancer survivors in the cancer registry system based on the Cancer Supplement of the Medical Expenditure Panel Survey.^29^, ^30^ The project was funded by the Rockefeller‐endowed China Medical Board, and the project covered an area of eight counties and districts to obtain a representative sample of the cancer survivors of Shandong province. In each county or district, about 200 cancer cases were randomly chosen from the local cancer registry. To improve the response rates, local health officials were hired to conduct this household survey and all them received a formal intensive training before the in‐person interviews. The project team attempted to contact 2600 patients, but 18% of them had died, 15.7% had relocated, 3.5% had provided the wrong address, and 0.5% declined to participate the survey. Finally, about 1600 respondents were interviewed and completed the questionnaire. The survey was conducted in 2015 and 2016. The more detailed study design has been published previously.^30^, ^31^ This study was approved by the Human Research Ethics Committee at the School of Public Health, Shandong University (20140201). Written informed consent was obtained from all participants before starting the interviews.

### 2.2 Participants

**Figure A1:**
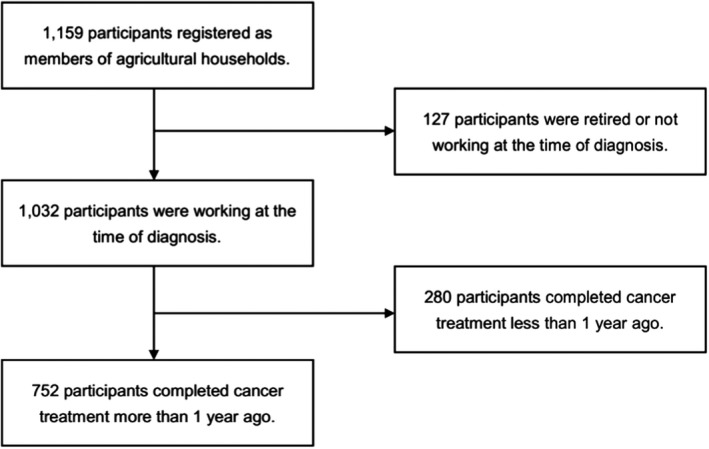
Criteria for inclusion in study

Patients included in this study were: (a) 18‐79 years at the time of cancer diagnosis; (b) diagnosed with colorectal, lung, stomach, or female breast cancers during 2011‐2013; (c) registered as members of agricultural households^12^; (d) worked as farmers or employees at the time of diagnosis; and (e) had completed primary cancer treatments (surgery, chemotherapy, and radiation therapy) more than 1 year ago. In addition, patients with a secondary cancer were excluded. A total of 752 cancer survivors were ultimately analyzed in this study (Figure A1).

### 2.3 Measures

We collected data including the date of diagnosis, type of cancer, cancer stage, and age at diagnosis from the cancer registry system. The questionnaire was used to collect information on sex, marital status, educational level, household income, residence registration, job/industry type, health insurance type, and the presence of other chronic diseases.

We assessed changes in employment and income by asking the participants several questions, such as “One year after completing the cancer treatment, did you shorten your working hours because of your cancer, its treatment or the lasting effects of the treatment?” and “One year after completing the cancer treatment, was your income (wages, agricultural income, rent, pensions, stock dividends, interest, and social security) reduced because of cancer, its treatment or the lasting effects of the treatment?” The informal caregivers' work and income changes were assessed with similar questions. These questions all had the same answer options: increased; no change; reduced by less than half; reduced by half; reduced by more than a half; stopped working/no longer have any income. We asked the participants to assess “whether cancer, its treatment or the lasting effects of the treatment have impaired your physical or mental work ability.”

### 2.4 Statistical analyses

Descriptive statistics were used to describe the distribution of sociodemographic and clinical characteristics as well as the work‐related outcomes of the sample population. The work changes were divided into the following three groups in the univariate analysis: (a) no change; (b) reduced working hours; and (c) stopped working or farming. A chi‐square test was conducted to assess the difference in work change status among the sociodemographic and clinical subgroups. We also analyzed the ability to perform physical tasks among different groups of survivors based on their change in work status; Bonferroni correction was then used to correct the experiment‐wise error rate when using multiple comparisons.

We defined the dependent variable as a dichotomous variable (stopped working vs continued to work) and then used logistic regression models to identify the effect of the various variables on work changes following cancer treatment. Two logistic regression models were constructed by using the data from the female group and the male group; in both cases, the dependent variable was whether the patient stopped working or not. Because of the significant collinearity between job type and health insurance type, only job type was included in the models. All statistical analyses were performed by SPSS, Version 21. All statistical tests in this study were two‐sided (*P* < .05).

## 3 RESULTS

### 3.1 Characteristics of rural cancer survivors

Table 1 below presents the detailed sociodemographic and clinical characteristics for participants. A majority of the cancer survivors was female (56%), and nearly 70% of them were less than 65 years old. Nearly all participants (96%) were farmers covered by rural health insurance. Just over half of the participants had an education level less than a middle school degree. Nearly two‐thirds of the participants had annual household incomes of 20 000 CNY or lower. The predominant cancer sites were breast and stomach in women, and lung and colorectal in men. Sixty‐seven percent of survivors completed cancer treatment (surgery, chemotherapy, and radiation therapy) more than 2 years ago.

**Table 1:** Participant characteristics (n = 752)

Characteristics	N	%
Sex		
Male	331	44.0
Female	421	56.0
Age at time of survey		
≤40	29	3.9
41‐55	221	29.4
56‐65	268	35.6
>65	234	31.1
Marital status		
Not currently married	64	8.5
Married	688	91.5
Employment status before cancer diagnosis		
Farmers	718	95.5
Others	34	4.5
Education		
Did not finish primary school	128	17.0
Finished primary school	320	42.6
Finished middle school	265	35.2
Finished high school or above	39	5.2
Health insurance type		
New rural cooperative medical insurance	702	93.4
Other	50	6.6
Yearly family income		
<5000 CNY^a^	137	18.2
5000‐19 999 CNY	340	45.2
20 000‐50 000 CNY	231	30.7
>50 000 CNY	44	5.9
Comorbidity		
0	570	75.8
≥1	176	23.4
Did not answer	6	0.8
Cancer site		
Colorectal	229	30.5
Female breast	233	31.0
Lung	109	14.5
Stomach	181	24.0
Cancer stage at diagnosis		
0‐I	268	35.6
II	300	39.9
III‐IV	57	7.6
Missing	127	16.9
Time since completed treatment		
1‐2 y	246	32.7
>2 y	506	67.3

a 10 000 Chinese Yuan≈1541 US dollars as of 31 December 2015; per capita disposable income of Chinese rural residents in 2015 was 11 421 Chinese Yuan.

### 3.2 Work‐related outcomes among rural cancer survivors and their caregivers

**Figure 1:**
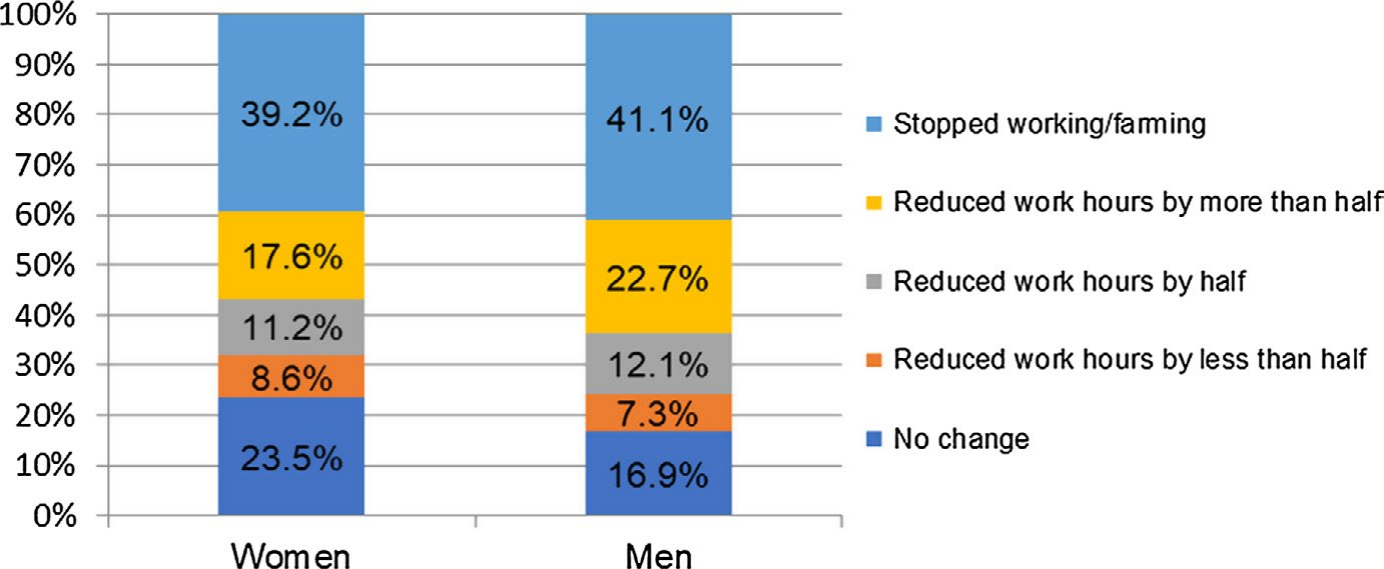
Changes in work hours among cancer survivors

**Figure 2:**
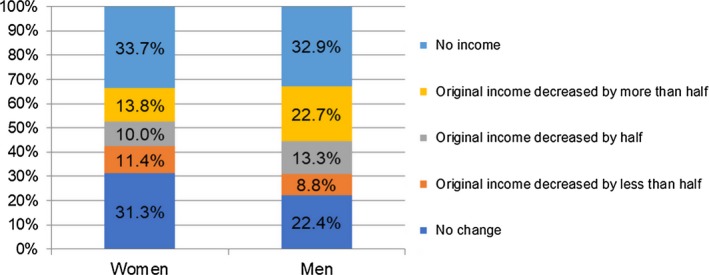
Changes in income among cancer survivors

**Figure 3:**
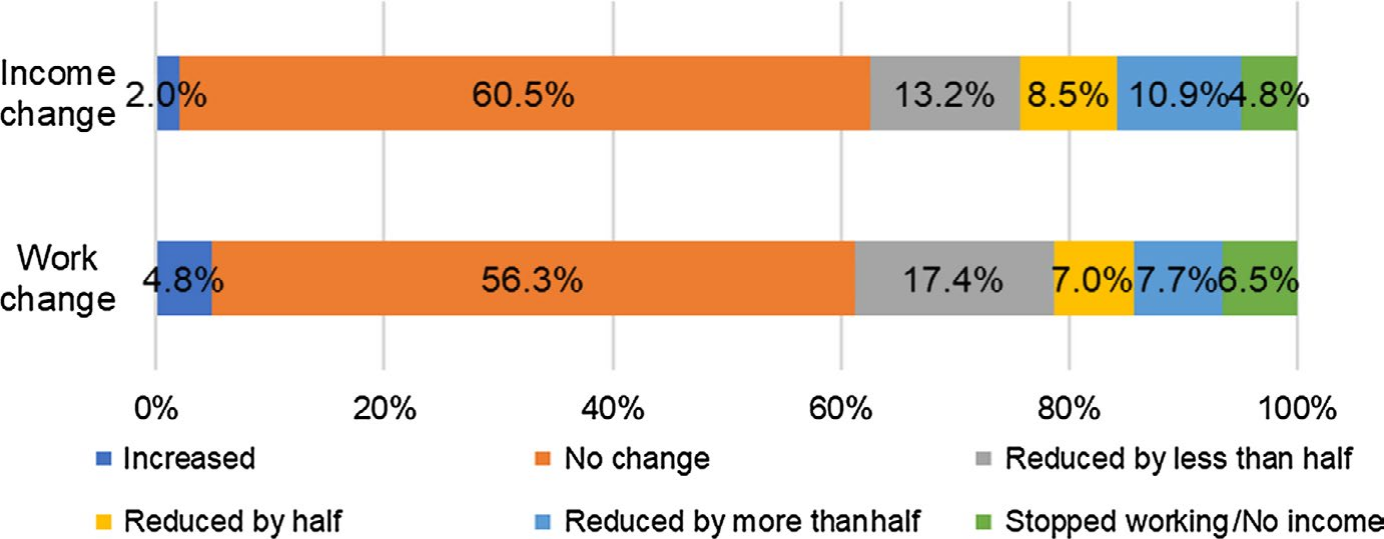
Changes in work hours and income among informal caregivers

Figures 1 and 2 show the work and income changes of cancer survivors 1 year after completing cancer treatment, respectively. Among 421 female cancer survivors, 24% reported keeping their regular working hours; 37% reduced their working hours; and 39% of them stopped working/farming due to cancer and its treatments. Among 331 male cancer survivors, 17% reported keeping their regular working hours; 42% reduced their working hours; and 41% of them stopped working/farming due to cancer and its treatments. The income changes of cancer survivors 1 year after completing cancer treatment were similar to the work changes. Among 752 participants, about 34% of women and 33% of men reported that they had no source of income 1 year after completing cancer treatment. As for informal caregivers, as a result of taking care of those with cancer, 44% of them reported a change in employment status, and 34% of them witnessed a decrease in their income (shown in Figure 3).

### 3.3 Ability to perform physical tasks for rural cancer survivors

**Figure 4:**
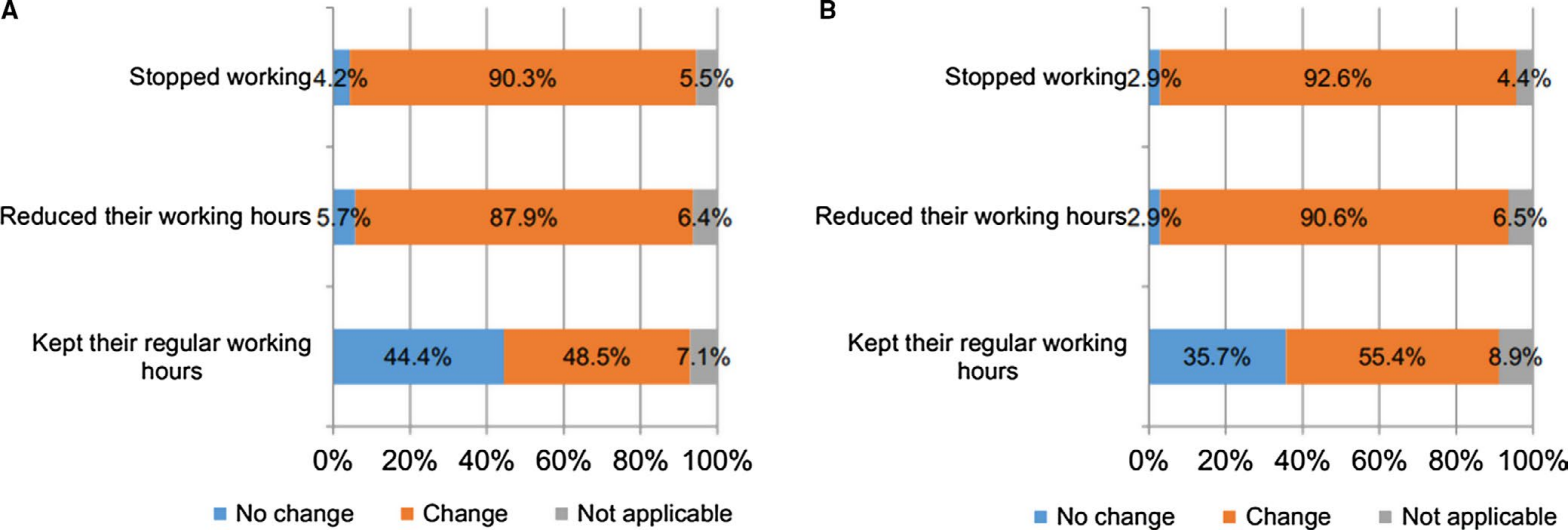
After treatment, did cancer interfere with your ability to perform any physical tasks required by your job? A, Women; B, men (There are three work groups (stopped working, reduced work hours, and no change in work hours) by their ability to perform physical tasks.).

Figure 4 breaks down the three work groups (stopped working, reduced work hours, and no change in work hours) by their ability to perform physical tasks. Even though half of the 155 participants who maintained their regular working hours reported that their ability to do physical work had decreased, they still kept the same working hours as before. Among cancer survivors who kept their regular working hours, 44% of women and 36% of men reported that their ability to perform physical tasks had not been impaired. The majority of those who reduced their working hours or stopped working altogether reported physical ability limitations as a result of cancer. Only 22 (3%) respondents among all 752 respondents reported that they did not need to perform any physical tasks during work.

Multiple comparisons of the three groups regarding their ability to perform physical tasks show that the participants who kept their regular working hours differed from those who reduced working hours and stopped working (*P* < .001). However, there was no statistically significant difference between participants who reduced working hours and those who stopped working.

### 3.4 Factors influencing the decision to stop working

Table 2 summarizes the results from the multivariable analysis of the factors that influence the decision to stop working. Among women, survivors with a middle school education level were less likely to report leaving the workforce than those who did not finish primary school (odds ratio [OR] = 0.35, 95% CI: 0.16‐0.77). Patients with no physical ability limitations were also less likely to stop working compared to those who did have physical limitations at work (OR = 0.19, 95% CI: 0.08‐0.45). Additionally, female stomach cancer patients were more likely to not return to work compared to colorectal cancer patients (OR = 2.74, 95% CI: 1.12‐6.74). There were no significant differences in work change status by age group, income, marital status, employment type, comorbidity, cancer stage, or duration of disease since diagnosis.

**Table 2:** Logistic regression analyses evaluating the association of independent factors with an end in work

	Women		Men	
	OR (95% CI)	*P*‐value	OR (95% CI)	*P*‐value
Age at time of survey^a^	1.02 (0.99‐1.05)	.19	1.06 (1.02‐1.10)	<.01
Marital status				
Not currently married	Ref		Ref	
Married	0.47 (0.17‐1.29)	.14	0.23 (0.07‐0.77)	.02
Employment status before cancer diagnosis				
Farmer	Ref		Ref	
Others	0.85 (0.22‐3.28)	.82	0.47 (0.06‐3.58)	.47
Education				
Did not finish primary school	Ref		Ref	
Finished primary school	0.65 (0.33‐1.28)	.22	0.23 (0.08‐0.63)	<.01
Finished middle school	0.35 (0.16‐0.77)	<.01	0.29 (0.10‐0.86)	.03
Finished high school or above	0.45 (0.10‐2.03)	.30	0.58 (0.11‐3.11)	.53
Yearly family income				
<5000 CNY	Ref		Ref	
5000‐19 999 CNY	1.29 (0.63‐2.62)	.49	0.68 (0.30‐1.54)	.36
20 000‐50 000 CNY	1.12 (0.53‐2.35)	.77	0.77 (0.31‐1.94)	.58
>50 000 CNY	2.48 (0.72‐8.49)	.15	9.67 (1.56‐59.85)	.02
Comorbidity				
0	Ref		Ref	
≥1	0.83 (0.46‐1.48)	.52	0.90 (0.45‐1.78)	.76
Cancer site				
Colorectal	Ref		Ref	
Female breast	2.09 (0.95‐4.60)	.07	—	—
Lung	0.95 (0.32‐2.82)	.93	2.96 (1.37‐6.40)	<.01
Stomach	2.74 (1.12‐6.74)	.03	1.28 (0.65‐2.53)	.48
Cancer stage at diagnosis				
0‐I	Ref		Ref	
II	0.90 (0.54‐1.49)	.68	2.23 (1.17‐4.23)	.02
III‐IV	0.57 (0.20‐1.61)	.29	0.72 (0.25‐2.09)	.54
Time since completed treatment				
1‐2 y	Ref		Ref	
>2 y	0.97 (0.57‐1.66)	.91	0.79 (0.43‐1.45)	.44
Physical work limited				
Yes	Ref		Ref	
No	0.19 (0.08‐0.45)	<.001	0.23 (0.06‐0.89)	.03

a Age was entered as a continuous variable in the regression model.

Among male cancer survivors, participants who were older (OR = 1.06, 95% CI: 1.02‐1.10), or had an annual household income of more than 50 000 Chinese Yuan (OR = 9.67, 95% CI: 1.56‐59.85) were more likely to report leaving the workforce. Participants who were married tended to continue to work compared to those who were not married (single, widowed, or divorced), with an OR value of 0.23 (95% CI: 0.07‐0.77). Participants with a primary school (OR = 0.23, 95% CI: 0.08‐0.63) or middle school education (OR = 0.29, 95% CI: 0.10‐0.86) were less likely to report no longer working than those who did not finish primary school. Lung cancer patients were more likely to stop working compared to colorectal cancer patients (OR = 2.96, 95% CI: 1.37‐6.40). Patients with no physical ability limitations were less likely to stop working compared to those with physical ability limitations (OR = 0.23, 95% CI: 0.06‐0.89).

## 4 DISCUSSION

The main objective of this study was to describe cancer‐related work outcomes among rural Chinese cancer survivors. Although the significance of employment for cancer survivors has emerged in prior studies, the majority of these studies focused on urban cancer employees with a favorable psychosocial work environment and support service.^5^, ^6^, ^7^, ^8^ The results of our study provide evidence of occupational problems among cancer survivors who reside in rural China. In our study, approximately 40% of rural cancer survivors reported that they stopped working or farming due to cancer, which is much higher than that of another study conducted in rural America (19%).^32^ Our study also showed that 39% of rural cancer survivors reduced their work hours; again, this proportion is higher than that of another study conducted in America (31%).^24^ These occupational issues are profound as many rural cancer survivors are forced to rely on their limited savings to support themselves or rely on the support of their family members.

Our study has shown that rural survivors were less likely to continue to work after cancer in China. There are several possible factors that may result in rural cancer survivors leaving the work force altogether or reducing their work hours. First, holding multiple jobs is the typical work pattern in rural China.^33^ Most peasants, alongside their farm business, are engaged in nonagrarian (eg, transport, industry, mining, and trading) activities to supplement their income.^13^ Shifting between these two forms of work—agrarian and nonagrarian—can occur on a seasonal or even daily basis.^33^ Carlsen et al have reported that manual labor is a risk factor for unemployment among cancer survivors.^34^ This certainly applies to the participants in our study as both the agrarian  and  nonagrarian activities typical of Chinese rural populations classify as manual labor, with long hours and high intensity, making it difficult for farmers to return to work.^12^ To exacerbate this issue, many peasant migrant workers work without sufficient legal protection. The current rural labor market in China exists in a legal vacuum, and the labor relations of agricultural employees are almost completely unprotected by the labor legal system under the current law.^15^ Adjusting relevant labor laws and regulations for rural cancer survivors to develop feasible and sustainable workplace policies are urgently needed. Second, related studies have indicated that lower health‐related quality of life (HRQoL) was negatively associated with the employment status of cancer survivors.^35^ Although this study did not explore the relationship between return to work and quality of life, our previous study found that rural cancer survivors have impaired HRQoL and a substantial proportion (59%) of patients reported pain/discomfort and 39% reported psychological problems.^36^ These factors—higher intensity work and lower HRQoL—may explain the high proportion of rural cancer survivors choosing to stop working.

Our study also found that cancer patients with a lower education level were more likely to stop working or farming, a noteworthy finding since low education levels, and the often resulting lower income levels, are typical characteristics of rural populations in China.^37^ An anthropological study of Chinese “cancer villages” revealed that residents usually thought that “there [was] no cure for cancer, once you get it, you can only wait to die”.^38^ This attitude and an overall lack of awareness of cancer may explain the large proportion of patients reporting a negative effect on their employment status. Additionally, compared with low‐income families, male patients whose household income was more than 50 000 Yuan were more likely to stop working. Those rural cancer survivors who had more income may have been able to stop working because they had sufficient savings, while those with less money may have felt obligated to return to workforce. However, we found no significant association between household income level and women leaving the workforce. It may be related to the traditional gender concept in rural China that men play the key role in making money, while women mainly play an important role in raising children, supporting the elderly and performing household duties.^39^

Our study found that not only did cancer survivors face significant challenges in returning to work, but their caregivers experienced employment difficulties as well. Forty‐four percent of family members' employment status changed, and 7% stopped working altogether to provide care even 1 year following the completion of treatment. This is comparable to de Moor's study, which found that 25% of cancer survivors' caregivers made extended employment changes and 12% of caregivers were not employed.^40^ This burden often falls on family caregivers in rural areas because of their flexible work schedules,^33^ allowing them to more easily reduce their work hours, and subsequently causing them to face a decline in income. However, extant studies on informal cancer caregivers in China have mainly focused on their psychological changes and physical health problems. Few studies have addressed employment issues, such as the loss of a family's main source of income, reduced work hours for caregivers, and the loss of opportunities for caregivers to participate in social and leisure activities.

Although the economic burden of cancer survivorship can be calculated by assessing medical costs and productivity loss,^41^ our descriptive data regarding changes in cancer survivors and their caregivers' income provides a general understanding of their economic conditions. Even though cancer patients may have insurance programs for catastrophic diseases or medical assistance,^16^, ^42^ our study has shown that the consequences of cancer do not end following treatment. In fact, surviving cancer poses a threat to their families' financial well‐being.

This was a descriptive, retrospective, cross‐sectional study describing the work and financial impact of cancer in rural China. Our main findings provide pivotal information for Chinese policymakers to reduce the burden of cancer, and help health‐care providers to recognize the importance of the ability to return to work for working age cancer survivors in rural areas. Unlike prior studies, we examined cancer survivors' employment status and influencing factors stratified by gender. Documenting the financial burden to family caregivers is another strength. Owing to the special cultural context in China, most Chinese cancer survivors mainly rely on their family members in the ensuing daily life, rather than hiring professionals. By describing work‐related outcomes among rural cancer family caregivers, we provided a fuller picture of cancer survivorship and its effects on the entire family.

Some limitations should be noted with our study. First, this study was based on a household‐reported survey. Potential recall and reporting biases may have occurred during the data collection stage. Second, data on work‐related outcomes were reported by the cancer patients. It is possible that cancer survivors underestimated or overestimated the work changes that their caregivers experienced. Third, the lack of a comparison group made up of the general population or of urban cancer survivors makes it impossible to assess whether there are any disparities between the rural survivors and the reference group with respect to the relationship between the explanatory variables and employment status. Finally, this is a cross‐sectional study, which cannot collect data on the change in survivors' employment over time. The survey asked about employment changes 1 year after completing cancer treatment, rather than employment changes that may have occurred at any time since diagnosis. We were not able to study short term work changes with the data. Further research should focus on the longitudinal impact of cancer survivorship on employment.

## 5 CONCLUSIONS

This study was a preliminary exploration of the changes in employment among cancer survivors in rural China. The results indicate that cancer survivors living in rural China, compared to those from other studies, had a high proportion of unemployment and work disability. Therefore, the government should develop a comprehensive supportive and survivorship care plan for rural cancer survivors. Cancer prevention and control strategies are needed to address the economic burden of cancer survivorship and broader aspects of well‐being. Health care providers should provide support and assistance for cancer survivors to return to work after cancer diagnosis. Lastly, it is necessary to incorporate employment concerns into their treatment and rehabilitation.

## CONFLICT OF INTEREST

The authors have no conflicts of interest to disclose.

## AUTHOR CONTRIBUTIONS

Mingzhu Su was involved in conceptualization, methodology, data curation, formal analysis, software, writing–original draft, and writing–review and editing. Nan Zhang and Yuanchu Cai were involved in investigation and data curation. Jialin Wang was involved in supervision, project administration, and resources. Roger Anderson was involved in supervision, project administration, and writing–review and editing. Nengliang Yao and Xiaojie Sun were involved in conceptualization, methodology, investigation, resources, supervision, project administration, and writing–review and editing. All authors approved the final version.

## ETHICAL APPROVAL

All procedures performed in this study are in accordance with ethical standards of the Institutional Review Board at the School of Public Health at Shandong University (no. 20140201), and with the Declaration of Helsinki.

## INFORMED CONSENT

Written informed consent was obtained from all participants before data collection.
